# Age-Related and Lifestyle-Related Differences in Gut Microbiota Composition Among Community-Dwelling Adults

**DOI:** 10.3390/microorganisms14081841

**Published:** 2026-08-19

**Authors:** Siyu Li, Xuefei Zhao, Minjia Chen, Yi Cheng, Qiwei Wu, Yangyang Feng, Ailin Yi, Lu Chen, Yuting Tian, Yanling Wei

**Affiliations:** Chongqing Key Laboratory of Digestive Malignancies, Department of Gastroenterology, Engineering Research Center for Advanced Medical Technology in Microecological Therapy of Chongqing, Daping Hospital, Army Medical University (Third Military Medical University), Chongqing 400042, China; lsylsy@tmmu.edu.cn (S.L.); xfzhao20@tmmu.edu.cn (X.Z.); chenmj525@tmmu.edu.cn (M.C.); chengyidph@tmmu.edu.cn (Y.C.); wqw123456@tmmu.edu.cn (Q.W.); youngyangfeng@tmmu.edu.cn (Y.F.); yalyal120@tmmu.edu.cn (A.Y.); chenlu130@tmmu.edu.cn (L.C.); tianyuting@tmmu.edu.cn (Y.T.)

**Keywords:** gut microbiota, lifestyle, age, 16S rRNA sequencing

## Abstract

As global aging accelerates, identifying age- and lifestyle-associated gut microbiota alterations has become critical for understanding healthy aging and microbiota-targeted interventions. To explore the associations of age and lifestyle factors with gut microbiota profiles in community-dwelling adults, 159 Chongqing residents were divided into young (18–39, *n* = 44), middle-aged (40–59, *n* = 71) and elderly (60–89, *n* = 44) groups. Fecal samples were analyzed by 16S rRNA gene sequencing, and lifestyle factors were assessed using a study-specific structured questionnaire. The Shannon index differed significantly between the young and middle-aged groups (*p* < 0.05), whereas other α-diversity and β-diversity analyses showed no significant differences. Age-related shift trends included decreased *Bacillota* and increased abundance of *Bacteroidota*. Young adults were enriched in *Parasutterella*, the *[Eubacterium] ventriosum* group and *Anaerostipes*; middle-aged adults were enriched in *Clostridium* sensu stricto 4, *Clostridium* sensu stricto 1 and *Holdemanella*; and elderly adults were enriched in *Prevotellaceae* UCG-004, *Helicobacter*, *Lactobacillus*, *Streptococcus* and *Fusobacterium*. Lifestyle factors were independently associated with specific microbial signatures, including tea-related enrichment of *Catenibacterium* and *Escherichia–Shigella*, exercise-related enrichment of *Dialister* and *Bavariicoccus*, and milk-related enrichment of *Bacillus* and *Klebsiella* (formerly *Raoultella*). Overall, these findings provide population-based evidence linking age and lifestyle factors with gut microbial profiles and may inform microbiota-targeted strategies for healthy aging.

## 1. Introduction

Gut microbiota refers to the microbial community colonizing the gut tract, with a total cell count on the order of 10^14^, which forms a vital “hidden organ” of the human body [1]. Starting from birth, an individual’s unique microbial community configuration is shaped by multiple determinants, including delivery mode, feeding patterns, environmental exposure and genetic background. Throughout the lifespan, the composition of gut microbiota is continuously modulated by dietary habits, lifestyle, living surroundings, pharmaceutical intervention, disease status and psychological conditions [2].

Aging is accompanied by impaired gut barrier function, altered gut architecture, and disrupted microbial homeostasis [3,4]. These age-related changes are associated with an increased risk of digestive dysfunction, gastrointestinal malignancies, and chronic inflammatory disorders in older adults [4,5]. Previous research has documented age-dependent declines in short-chain fatty acid (SCFA)-producing taxa such as *Roseburia intestinalis*, *Akkermansia* and *Faecalibacterium*, whereas the abundance of *Blautia hansenii* increases with advancing age [3]. Importantly, age-related gut microbial alterations appear to be at least partially modifiable. Accumulating evidence from human studies and animal models suggests that microbiota-targeted interventions, particularly probiotic supplementation, have the potential to support healthy aging by modulating gut microbial homeostasis, strengthening intestinal barrier function, attenuating inflammation, and improving age-related functional decline [6,7,8].

Besides chronological aging, lifestyle factors are also important modifiable determinants of gut microbiota composition. Previous studies have demonstrated that dietary habits and behavioral patterns can independently shape microbial communities and their metabolic potential. For example, tea polyphenols can be metabolized by the gut microbiota and may promote the growth of beneficial bacteria, including *Bifidobacterium* and *Akkermansia*, while suppressing potentially pathogenic taxa such as *Enterococcus* and *Streptococcus* [9,10]. Regular physical activity has been associated with greater microbial diversity and enrichment of SCFAs-producing bacteria [11]. Milk contains lactose, oligosaccharides, proteins, and other nutrients that can be utilized by gut microorganisms and may influence the abundance and metabolic activity of commensal bacteria [12]. These findings suggest that both aging and lifestyle factors may independently contribute to variations in gut microbial profiles.

Moreover, most studies have focused on specific populations or single lifestyle behaviors, limiting a comprehensive understanding of how age stratification and multiple lifestyle factors are independently associated with gut microbiota composition within the same community-based population. Accordingly, 159 community-dwelling adults, stratified into young (18–39 years), middle-aged (40–59 years), and elderly (60–89 years) groups, were enrolled, and their gut microbial landscapes were characterized via 16S rRNA gene sequencing to investigate the independent associations of age and lifestyle factors with gut microbiota composition. Our findings are expected to provide population-based evidence for understanding age- and lifestyle-related microbial patterns and inform future strategies for maintaining gut microbial homeostasis and promoting healthy aging.

## 2. Materials and Methods

### 2.1. Study Participants

This study recruited permanent residents of Chongqing from December 2024 to February 2025 through Daping Hospital, Army Medical University, Chongqing, China. In total, 162 individuals were initially screened, and 159 eligible participants were finally enrolled in the study. All enrolled subjects were stratified into three age subgroups: young group (YG, 18–39 years, *n* = 44), middle-aged group (MG, 40–59 years, *n* = 71), and elderly group (EG, 60–89 years, *n* = 44). Detailed inclusion criteria are as follows: (1) aged 18–89 years with any gender; (2) only participants who had not taken antibiotics, probiotics, prebiotics, or other medications and health supplements that may alter the gut microbiota within the previous 4 weeks [13,14,15]; (3) no family history of hereditary diseases; (4) female participants with a non-pregnant and non-lactating status; (5) free of infectious, contagious, or parasitic diseases; (6) no anemia, renal diseases, cardiovascular diseases, chronic inflammation, psychiatric disorders, or neurological disorders; (7) no hematological diseases or autoimmune visceral diseases complicated by immune dysfunction; (8) no current or previous history of malignant tumors, congenital malformations, degenerative lesions, or chromosomal abnormalities; (9) absence of severe gastrointestinal diseases including gastrointestinal perforation and inflammatory bowel disease; (10) free of severe geriatric illnesses; (11) no history of severe hypersensitivity; (12) no prior receipt of fecal microbiota transplantation; (13) no participation in other clinical studies 4 weeks prior to enrollment. All participants provided written informed consent. Information on previous diseases, medication use, and health status was collected by self-reported questionnaires. The study was conducted in accordance with the Declaration of Helsinki. The protocol was approved by the Ethics Committee of Daping Hospital, Army Medical University (Third Military Medical University) (Ethics Approval No. MR-50-25-005694). This was registered with the Chinese Clinical Trial Registry (ChiCTR2500097557).

### 2.2. Sample Collection

Participants were instructed to collect the first stool sample of the day to minimize potential diurnal variation and avoid urine contamination. All participants were required to stop using antibiotics, probiotics, prebiotics, and other microbiota-related supplements at least 1 month before fecal sampling. A sterile, general-purpose stool collection tube without any preservative buffer or stabilizing reagent was used for sample collection. Approximately 1–3 g of stools from the central portion was aliquoted into three separate tubes, minimizing air exposure. All samples were self-collected and transported to the laboratory in an insulated ice box. Within 1 h of collection, the samples were transferred by study personnel and immediately stored at −80 °C until DNA extraction.

### 2.3. Lifestyle Questionnaire

A study-specific questionnaire was developed based on previous epidemiological and gut microbiome studies to assess habitual tea consumption, physical activity, and liquid milk intake (excluding yogurt and other dairy products) during the preceding six months. The questionnaire was designed with reference to frequency-based lifestyle assessments reported in previous studies. No dietary or lifestyle interventions were implemented before participant enrollment or fecal sample collection, and participants maintained their usual habits throughout the study.

Tea consumption and liquid milk intake were categorized as non-consumers (non-tea and non-milk groups) or tea/milk consumers (tea drinkers/milk consumers) according to the frequency of intake, with consumption defined as ≥1 time/week. Physical activity was classified according to the frequency of exercise sessions lasting >20 min as no exercise (non-exercise group) or exercise (exercise group), with regular exercise defined as ≥1 time/week. Information on tea preparation, milk type, and exercise intensity was not collected. The complete questionnaire is provided in Supplementary Table S1.

Participants reporting any tea consumption, milk consumption, or physical activity were assigned to the corresponding exposure groups, whereas those reporting none were assigned to the reference groups. The frequency categories and dichotomization strategy were based on published studies and are consistent with approaches commonly used in gut microbiome research [16,17,18,19].

### 2.4. Assessment of Participant Characteristics and Potential Confounders

Participant characteristics and potential confounding factors were assessed, including gastrointestinal symptoms, frailty status, and body mass index (BMI). Gastrointestinal symptoms were evaluated using the validated Gastrointestinal Symptom Rating Scale (GSRS), which consists of 15 items covering five symptom domains, including abdominal pain, reflux, indigestion, diarrhea, and constipation [20]. Frailty status was assessed using the Fried frailty phenotype criteria proposed by Fried et al., which include five components: unintentional weight loss, exhaustion, weakness, slow walking speed, and low physical activity [21]. Body mass index (BMI) was calculated as weight (kg) divided by height squared (m^2^). According to the recommended criteria for Chinese adults, BMI was categorized as underweight (<18.5 kg/m^2^), normal weight (18.5–23.9 kg/m^2^), overweight (24.0–27.9 kg/m^2^), and obesity (≥28.0 kg/m^2^) [22].

### 2.5. 16S rRNA Gene Sequencing and Bioinformatic Analysis

Total genomic DNA was extracted from stool samples using the Magnetic Soil And Stool DNA Kit (DP712, Tiangen Biotech, Beijing, China) according to the manufacturer’s instructions. Purified DNA was quantified using a Qubit fluorometer (Invitrogen, Carlsbad, CA, USA) and stored at −20 °C until further analysis. Specific primers targeting the V3-V4 hypervariable region of the 16S rRNA gene were applied: 341F (5′-CCTACGGGNGGCWGCAG-3′), 805R (5′-GACTACHVGGGTATCTAATCC-3′). The PCR was performed in a total reaction volume of 25 μL: DNA template at 5–50 ng, forward primer (1 μM) at 2.5 μL, reverse primer (1μM) at 2.5 μL, Pusion Hot start flex 2X Master Mix at 12.5 μL and finally ddH_2_O up to 25 μL. PCR conditions consisted of initial denaturation at 98 °C for 30 s, followed by 32 cycles of denaturation (98 °C, 10 s), annealing (54 °C, 30 s), and extension (72 °C, 45 s), with a final extension at 72 °C for 10 min. PCR products were purified with AMPure XP Beads (Beckman Coulter Genomics, Danvers, MA, USA) and quantified via Qubit (Invitrogen, USA). Qualified PCR products were evaluated using Illumina library quantification kits (Kapa Biosciences, Woburn, MA, USA). The amplicon library was paired-end-sequenced (2 × 250) on a DNBSEQ-G99 (LC-Bio Technology Co., Ltd., Hangzhou, China).

Subsequent microbiome bioinformatic analyses were performed with Quantitative Insights Into Microbial Ecology 2 (QIIME2, version 2025.7). Primer sequences were trimmed using the Cutadapt plugin in QIIME2 (version 2025.7), and duplicate reads were identified and removed via Vsearchplugin in QIIME2 (version 2025.7). The resulting high-quality reads were then processed for amplicon sequence variant (ASV) inference and taxonomic annotation using the DADA2 algorithm plugin in QIIME2 (version 2025.7). The R package vegan (version 4.4.1) was used to calculate α-diversity, including Shannon, Observed, Simpson, and Chao1 indices. The β-diversity was evaluated based on the Bray–Curtis and Jaccard distance matrices. Principal coordinate analysis (PCoA) was performed for each distance matrix to visualize the clustering patterns of microbial communities across samples. Permutational multivariate analysis of variance (PERMANOVA) with 999 permutations was applied to evaluate the significance of community dissimilarity. The Wilcoxon rank-sum test or Kruskal–Wallis test was used to identify differences in genus abundance. Built-in R functions and the ggplot2 package (version 3.5.1) were utilized for all calculations and data visualization.

### 2.6. Statistical Analysis

Descriptive statistics were employed to summarize the demographic characteristics and lifestyle of the study population. Continuous variables were presented as the mean (standard deviation [sd]), categorical variables were presented as the median (interquartile range [IQR]), and binary data were presented as *n* (%). SPSS statistical software, version 26.0, was used for statistical analysis. The Kruskal–Wallis test was used to estimate differences between quantitative variables, while the categorical variables were compared using Pearson’s chi-square test.

## 3. Results

### 3.1. Study Participants and Clinical Characteristics

**Figure 1:**
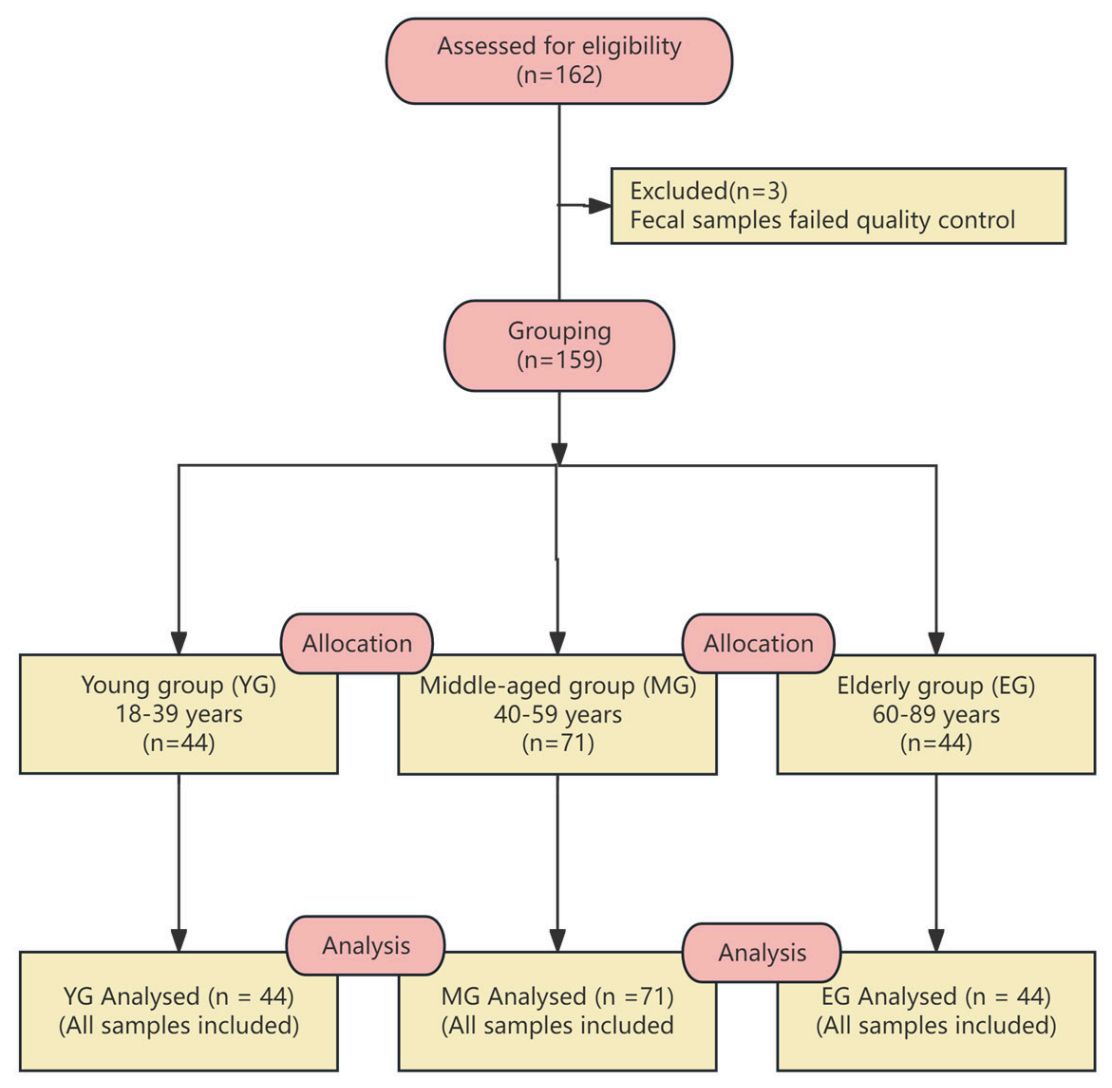
Flow chart of patients included and excluded in this trial.

As shown in Figure 1, from December 2024 to February 2025, 162 permanent residents were screened in Chongqing, and 159 individuals were finally enrolled, with permanent residences within a 150 km radius of Daping Hospital, Chongqing. Their median age was 52 (ranging from 38 to 60). Among them, 37.7% (*n* = 60) were male, 62.3% (*n* = 52) were female, 13.2% had a history of smoking, and 22.6% had a history of drinking. These participants were divided into the young group (YG, *n* = 44, 18–39 years), middle-aged group (MG, *n* = 71, 40–59 years) and elderly group (EG, *n* = 44, 60–89 years). The demographic and clinical information of the participants is shown in Table 1. There were no significant differences in gender, BMI stratification, frailty phenotype score, and GSRS among the three groups. However, exercise habits differed significantly among the groups, with the EG exhibiting a higher proportion of exercisers (48.2%).

### 3.2. Gut Microbial Diversity and Community Composition Across Age Groups

**Figure 2:**
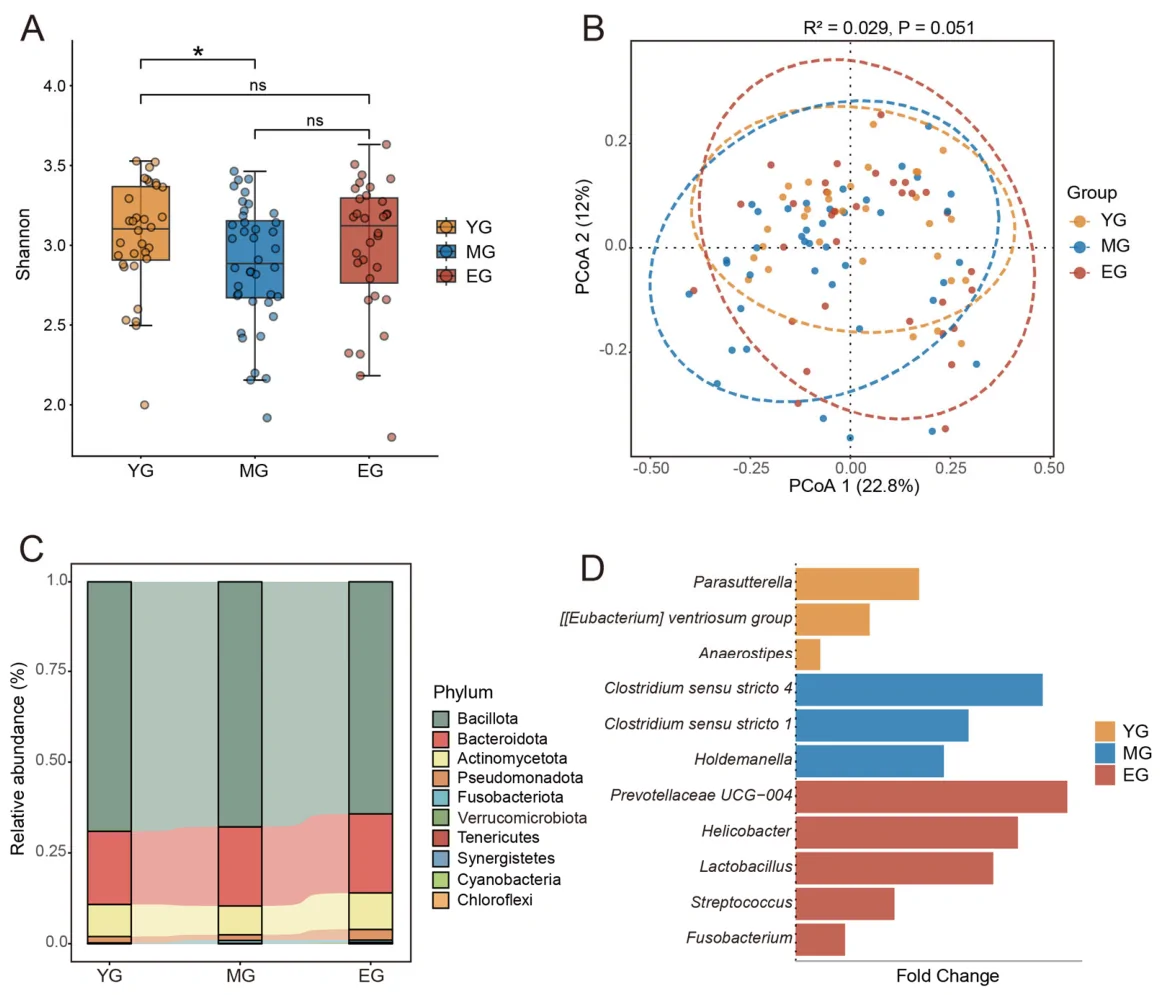
Associations between age and gut microbiota composition. (**A**) Shannon diversity index across the three age groups (YG: young group; MG: middle-aged group; EG: elderly group). Statistically significant differences were only observed between the young and middle-aged groups (*, *c* < 0.05; ns, not significant). (**B**) Principal coordinate analysis (PCoA) based on Bray–Curtis distance matrix. No significant overall differences in gut microbial community structure were detected among the three groups (PERMANOVA, R^2^ = 0.029, *p* = 0.051). (**C**) Stacked bar chart showing the relative abundance of gut microbiota at the phylum level. (**D**) Bar plot displaying the differentially abundant genera in each age group.

To characterize age-associated changes in gut microbial communities, we first compared α-diversity among the three age cohorts using the Shannon index. The results showed that the α-diversity of YG was significantly higher than MG, but there was no difference between YG and EG (Figure 2A). Compared with MG, the α-diversity of EG increased, but the difference was not statistically significant (Figure 2A). Next, we performed principal coordinate analysis (PCoA) based on the Bray–Curtis distance matrix to evaluate β-diversity. The analysis revealed that there was no overall difference in gut microbial community structures across the three age groups (Figure 2B). At the phylum level, the gut microbiota of the three groups was predominantly composed of *Bacillota* and *Bacteroidota*, followed by *Actinomycetota* and *Pseudomonadota*, with no gross compositional shifts observed across the three age groups (Figure 2C). Genus-level differential abundance analysis further identified distinct microbial signatures among groups (Figure 2D). We found that *Parasutterella, [Eubacterium] ventriosum* group and *Anaerostipes* were enriched in YG. The MG was characterized by elevated abundances of *Clostridium* sensu stricto 4, *Clostridium* sensu stricto 1 and *Holdemanella*. In contrast, genera such as *Prevotellaceae* UCG-004, *Helicobacter, Lactobacillus*, *Streptococcus* and *Fusobacterium* were significantly enriched in EG. These genus-level changes, though not accompanied by phylum-level restructuring, may point to age-responsive taxa that are worthy of further investigation.

### 3.3. Alterations in Gut Microbiomes Between Tea Consumers and Non-Consumers

**Figure 3:**
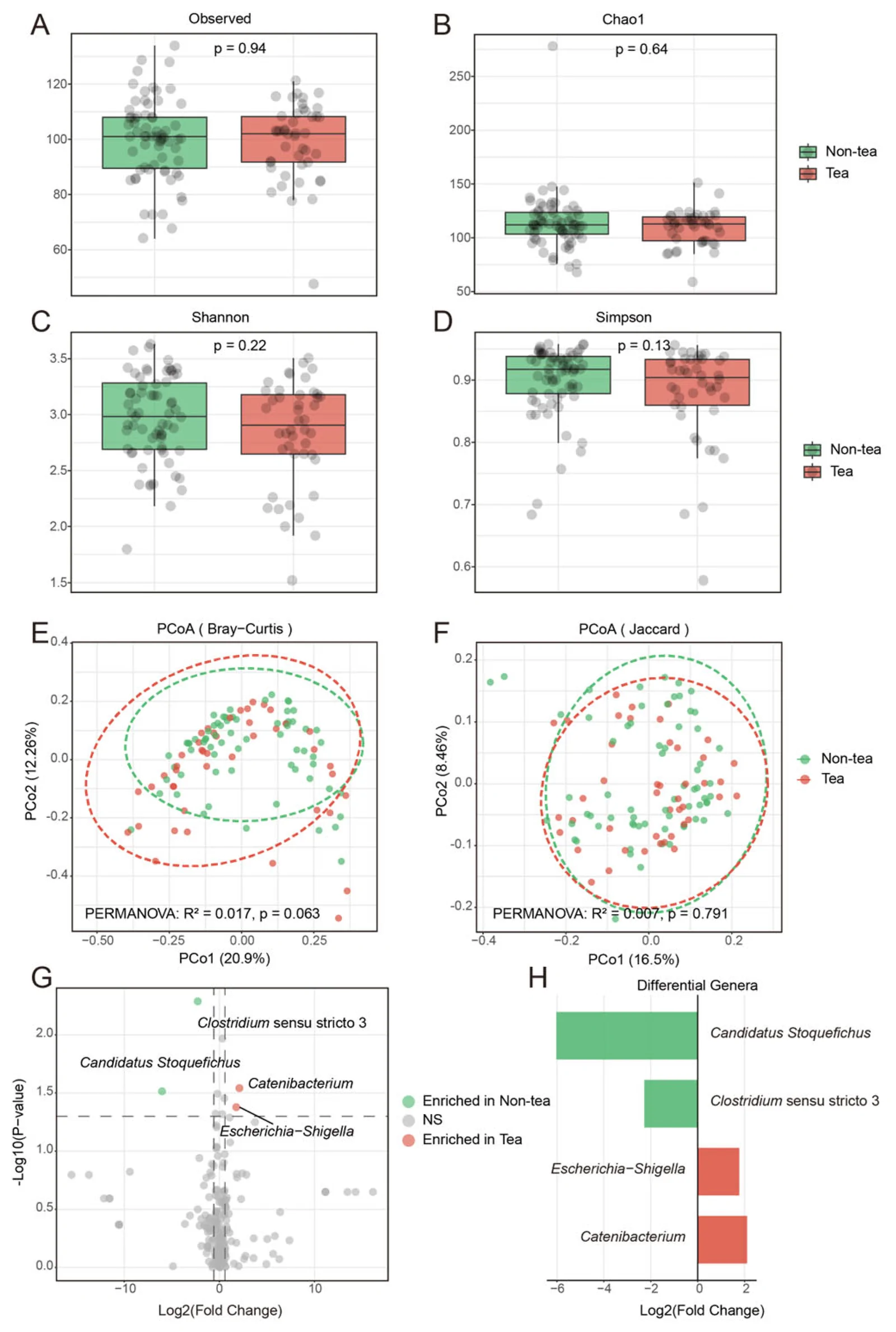
Gut microbial diversity and composition between tea consumers and non-tea consumers. Box plots of α-diversity indices comparing tea consumers (tea) and non-tea consumers (non-tea group), including observed species (**A**), Chao1 richness estimates (**B**), Shannon index (**C**), and Simpson index (**D**). No statistically significant intergroup differences were observed for any indices. PCoA of β-diversity based on Bray–Curtis distance matrix (**E**) and Jaccard distance matrix (**F**). PERMANOVA tests revealed no significant overall differences in microbial community structure between the two groups (Bray–Curtis: R^2^ = 0.017, *p* = 0.063; Jaccard: R^2^ = 0.007, *p* = 0.791). (**G**) Volcano plot displaying differentially abundant genera between two groups. (**H**) Bar plot of log_2_ (fold change) values for the four significant genera. Positive and negative values indicate enrichment in tea and non-tea groups, respectively.

We next investigated the association between lifestyle factors and gut microbial community structure, beginning with a comparison of *α*-diversity between habitual tea drinkers and non-tea consumers (non-tea group). The results showed that observed species, Chao1 richness estimates, and the Shannon and Simpson indices were not significantly different between the two groups (Figure 3A–D). In addition, PCoA based on Bray–Curtis and Jaccard distance matrices did not show obvious separation (Figure 3E,F), indicating that the microbial community structure was largely unaffected by tea-drinking status. Subsequently, we identified four genera that were significantly different between groups at the genus level. *Catenibacterium* and *Escherichia–Shigella* were enriched in tea drinkers, whereas *Clostridium* sensu stricto 3 and *Candidatus Stoquefichus* showed higher abundance in non-tea drinkers (Figure 3G,H).

### 3.4. Association of Physical Exercise with Gut Microbial Diversity and Composition

**Figure 4:**
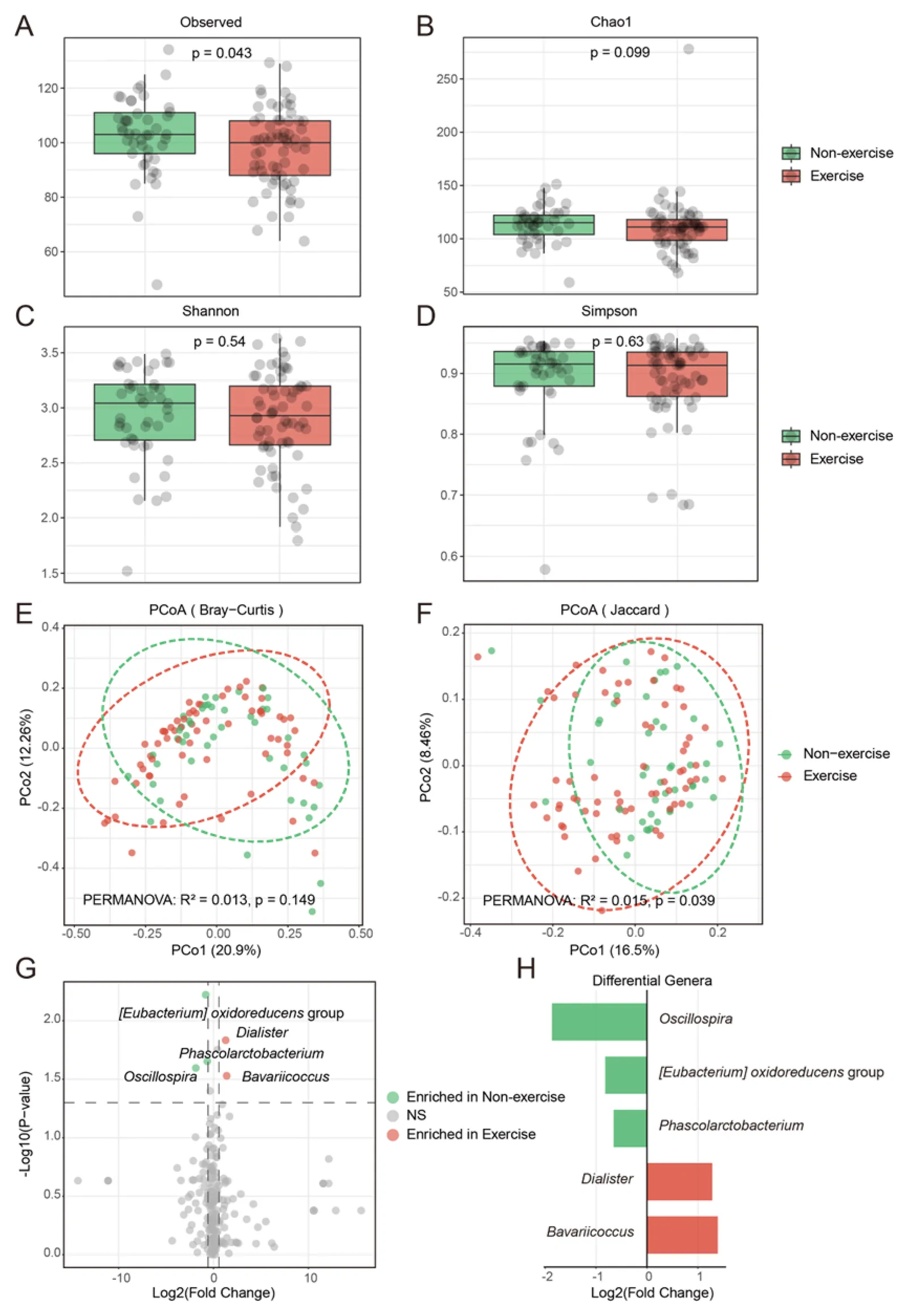
Gut microbial diversity and composition in relation to physical exercise habits. (**A**–**D**) Box plots of α-diversity indices for the exercise group and non-exercise group, including observed species (**A**), Chao1 richness estimates (**B**), Shannon index (**C**), and Simpson index (**D**). (**E**,**F**) Principal coordinate analysis (PCoA) of β-diversity calculated with a Bray–Curtis distance matrix (**E**) and Jaccard distance matrix (**F**). PERMANOVA tests revealed a marginal difference in overall microbial community structure between the two groups only under the Jaccard metric (Bray–Curtis: R^2^ = 0.013, *p* = 0.149; Jaccard: R^2^ = 0.015, *p* = 0.039). (**G**) Volcano plot displaying differentially abundant genera between two groups. (**H**) Bar plot showing significantly enriched genera in the non-exercise group (green) and exercise group (red).

Subsequently, we explored whether physical exercise habits are related to changes in gut microbiota. A comparison of α-diversity between the exercise and non-exercise groups showed that observed species were significantly elevated in the exercise group (Figure 4A), while the Chao1 richness estimates exhibited a slightly increasing trend (Figure 4B). The Shannon index and Simpson index (Figure 4C,D) did not differ significantly between the two groups. These results indicate that physical exercise may be related to species richness of the gut microbiota but may not affect evenness. β-diversity analysis based on Bray–Curtis distances revealed no significant separation between the exercise and non-exercise groups (Figure 4E). In contrast, analysis using Jaccard distances showed a significant although weak clustering by exercise status (*p* = 0.039, Figure 4F), suggesting that physical activity may influence the structure of the gut microbiota, albeit with a very small effect size. Differential microbial profiling was further performed to identify taxa changed by exercise status. Genera *Dialister* and *Bavariicoccus* were significantly enriched in individuals with physical exercise. In contrast, *Phascolarctobacterium*, *Oscillospira*, and the *[Eubacterium] oxidoreducens group* were increased in subjects lacking exercise habits (Figure 4G,H).

### 3.5. Effects of Milk Consumption on Gut Microbiota

**Figure 5:**
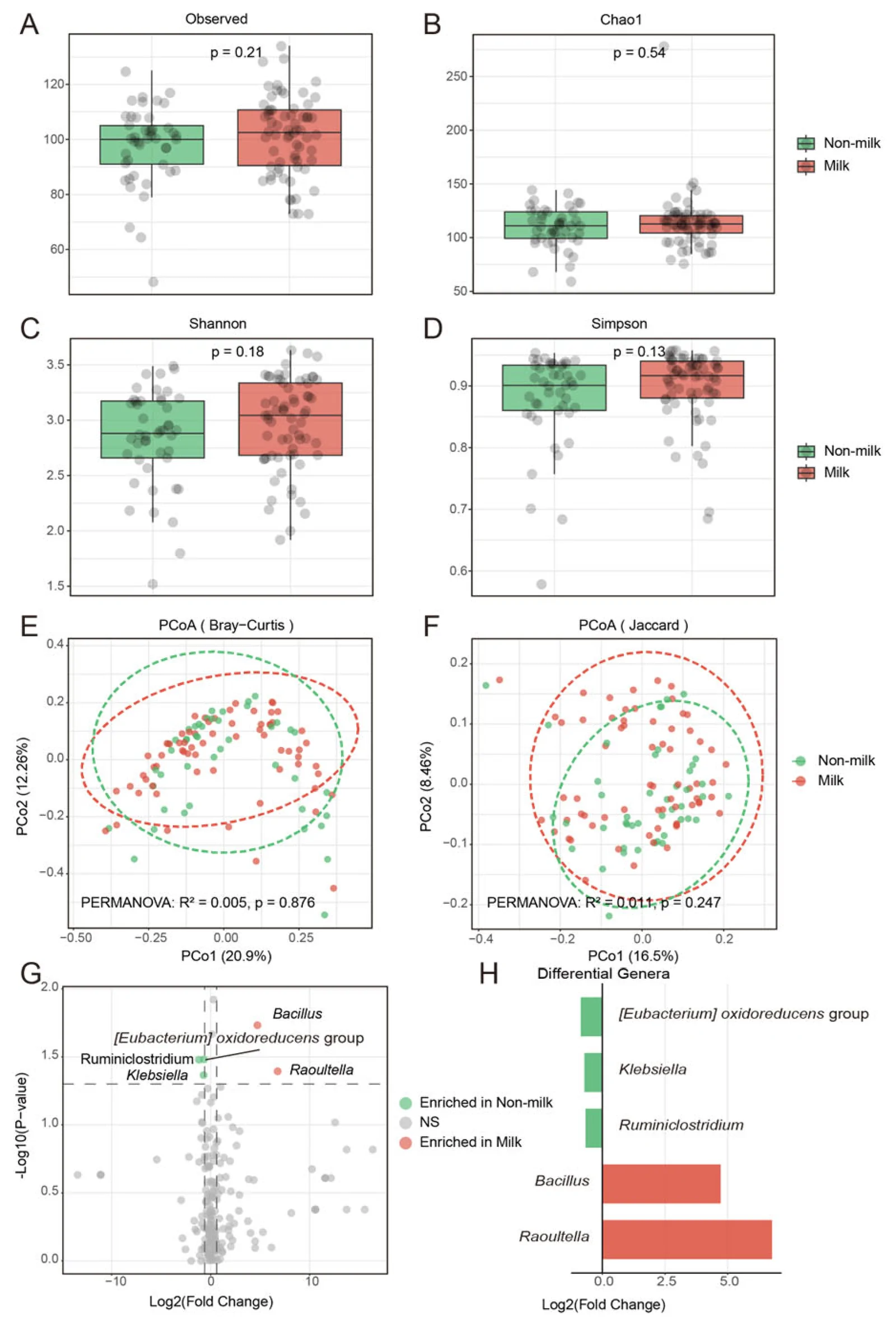
Gut microbial diversity and composition in relation to milk consumption habits. Box plots of α-diversity indices for the milk-intake group (milk) and non-intake group (non-milk group), including observed species (**A**), Chao1 richness estimates (**B**), Shannon index (**C**), and Simpson index (**D**). No statistically significant differences were identified between the two groups for all indices. PCoA of β-diversity based on Bray–Curtis distance matrix (**E**) and Jaccard distance matrix (**F**). PERMANOVA tests demonstrated no significant differences in overall gut microbial community structure between the two cohorts (Bray–Curtis: R^2^ = 0.005, *p* = 0.876; Jaccard: R^2^ = 0.011, *p* = 0.247). (**G**) Volcano plot displaying genus-level differential abundance between the milk and non-milk groups. (**H**) Bar plot showing significantly enriched genera in the non-milk (green) and milk (red) groups.

Next, we evaluated whether milk intake was associated with changes in gut microbes. The results showed that there was no significant difference in observed species, Chao1 richness estimates, and Shannon and Simpson indices between milk consumers and the non-milk group (Figure 5A–D). Furthermore, β-diversity analysis also showed no clustering separation trend between the two groups (Figure 5E,F). These results suggest that milk intake has little effect on the diversity and community structure of gut microbiota. Differential abundance analysis was similarly performed to identify microbes that differed between the two groups. Genera *Bacillus* and *Klebsiella* (formerly *Raoultella*) were enriched in the milk-consuming group, whereas the *Ruminiclostridium*, *[Eubacterium] oxidoreducens* group and other *Klebsiella* were significantly increased in the non-milk group (Figure 5G,H).

## 4. Discussion

Conventional theories have proposed that gut microbial α-diversity declines with advancing age [4]. Nevertheless, recent research demonstrates that α-diversity remains stable throughout adulthood in healthy individuals, with no significant distinctions between elderly and middle-aged/young populations [5]. The present study aligns with the findings of this observation. Discrepancies across published reports may stem from variations in sample size, age stratification criteria, and sequencing methodologies.

In our cohort, the relative abundance of *Bacillota* decreased progressively with age, whereas the abundances of *Bacteroidota*, *Actinomycetota* and *Pseudomonadota* increased. This age-associated taxonomic shift may reflect progressive alterations in gut microbial homeostasis during aging. The decline in *Bacillota*, which includes many SCFA-producing taxa, may suggest a potential reduction in microbial functions related to gut barrier maintenance and metabolic homeostasis, whereas the increased abundance of *Pseudomonadota* may reflect age-associated dysbiosis and increased inflammatory potential. Changes in *Bacteroidota* abundance may reflect altered dietary substrates and host physiological adaptation during aging. This pattern is broadly consistent with microbial compositional changes previously reported in aging populations [3].

Previous studies have reported reduced abundances of the butyrate-producing taxa *[Eubacterium] ventriosum* group and *Anaerostipes* during aging. Butyrate plays essential roles in maintaining epithelial barrier function, regulating immune responses, and suppressing inflammation [23,24]. Consistent with these reports, both taxa were enriched in the young participants in our study. Additionally, enriched in the young group, *Parasutterella* is a core commensal member of the gut microbiota and participates in bile acid metabolism, cholesterol metabolism, and purine metabolism [25]. The higher abundance of *Parasutterella* in the young group may reflect greater microbial metabolic potential. However, its biological functions appear to be context-dependent, as certain species have been reported to promote gut inflammation and colitis-associated colorectal cancer under pathological inflammatory conditions [26].

The middle-aged cohort was distinguished by characteristic enrichment of *Clostridium* sensu stricto 4, *Clostridium* sensu stricto 1 and *Holdemanella*, which have been associated with SCFA-related metabolic functions. Specifically, *Clostridium* sensu stricto 4 has been associated with Treg cell differentiation and IL-10-mediated anti-inflammatory responses [27], whereas *Clostridium* sensu stricto 1 and *Holdemanella* contain SCFA-producing members that have been linked to SCFA-related metabolic functions and favorable metabolic profiles, with several members exhibiting SCFA-producing potential [28,29]. Their enrichment in middle-aged adults may represent an age-associated microbial profile with enhanced SCFA-producing potential, which may contribute to the maintenance of gut homeostasis during aging.

Multiple genera, including *Prevotellaceae* UCG-004, *Helicobacter*, *Lactobacillus*, *Streptococcus* and *Fusobacterium,* were significantly enriched in elderly subjects. *Prevotellaceae* UCG-004 was enriched in the elderly group, although its biological function in humans remains poorly understood. Because members of the family *Prevotellaceae* are associated with carbohydrate and diet-related metabolism, this enrichment may reflect age-associated metabolic differences in the gut microbiota [30,31]. *Lactobacillus* was enriched in the elderly group in the present study. Previous studies have reported higher abundance of this genus in Moscow centenarians compared with conventional older adults [32]. In addition, specific *Lactobacillus* strains have demonstrated anti-aging effects in animal models by improving intestinal barrier function and alleviating inflammation and oxidative stress [33]. Furthermore, certain species within the genera *Streptococcus*, *Fusobacterium*, and *Helicobacter* have been associated with inflammaging and age-related disorders [34,35,36]. Collectively, our findings, together with previous evidence, support an association between aging and compositional shifts in the gut microbiota. However, because of the cross-sectional design, the present study cannot determine whether these microbial alterations are causally involved in the aging process or simply reflect age-related physiological changes.

Beyond chronological aging, lifestyle factors may also contribute to age-related variations in gut microbiota composition. Dietary habits, in particular, can modulate microbial metabolism and host–microbiota interactions. Therefore, we further investigated the relationship between lifestyle behaviors and microbial signatures in our cohort. Significant enrichments of *Catenibacterium* and *Escherichia–Shigella* were detected in tea drinkers. *Catenibacterium* has been associated with the fermentation of dietary polysaccharides, carbohydrate utilization, and SCFAs metabolism [37]. Unexpectedly, *Escherichia–Shigella* was enriched among tea drinkers in the present study. This finding differs from previous animal studies, in which tea was reported to suppress *Escherichia–Shigella* and pathogenic *Escherichia coli* [38,39]. The discrepancy may reflect differences between animal models and human populations, as well as the inability of genus-level 16S rRNA gene sequencing to distinguish pathogenic from non-pathogenic members of *Escherichia–Shigella*. In contrast, the gut microbiota of non-tea-drinkers was enriched with *Clostridium* sensu stricto 3 and *Candidatus Stoquefichus*. *Clostridium sensu stricto 3* was enriched in non-tea-drinkers in the present study. However, the biological significance of this finding remains unclear and warrants further investigation. Previous studies have reported that increased abundance of *Candidatus Stoquefichus* was negatively correlated with circulating TNF-α and IL-4 concentrations following dietary β-carotene supplementation, suggesting a potential association with anti-inflammatory responses [40]. Most existing studies linking tea consumption to gut microbiota have focused on distinct tea varieties. Variations in chemical constituents across tea types drive divergent shifts in microbial communities and subsequent health outcomes. Tea subtypes were not recorded in the present study, which may partially explain the discrepancies between our observations and findings from prior research.

*Dialister* and *Bavariicoccus* were significantly enriched in individuals with regular exercise. *Dialister* has been proposed as a movement-dependent microbial biomarker, and its abundance can be elevated by low-intensity routine physical activity [41]. In contrast, the non-exercise group exhibited enrichment of *Phascolarctobacterium*, *Oscillospira*, and the *[Eubacterium] oxidoreducens* group. *Phascolarctobacterium* shows a strong negative correlation with physical activity levels and accumulates abundantly in sedentary populations with low daily activity [42]. As a butyrate-producing taxon, *Oscillospira* is positively associated with metabolic health and intact gut barrier function [43]. Notably, neither the *[Eubacterium] oxidoreducens group* nor *Bavariicoccus* has been reported in previous exercise-related investigations, and human clinical evidence regarding these two genera remains scarce to date.

*Bacillus* and *Klebsiella* (formerly *Raoultella*) were significantly enriched among milk consumer subjects. *Bacillus* is widely recognized as a probiotic genus and has been identified as a core member of the healthy bovine milk microbiota, where it is commonly detected at relatively high abundance [44]. In contrast, members of the genus *Klebsiella* (formerly *Raoultella*) include opportunistic pathogens [45]. Therefore, the enrichment of *Klebsiella* (formerly *Raoultella*) in milk consumers should be interpreted with caution. Its association with habitual dairy intake remains unclear and warrants further investigation. Individuals who did not consume milk were enriched with *Ruminiclostridium*, *[Eubacterium] oxidoreducens* group, and *Klebsiella.* Previous animal studies have reported altered abundance of *Ruminiclostridium* in high-fat-diet-induced metabolic disorders, suggesting a potential association with host metabolism. However, its relevance in humans remains unclear [46,47]. The proliferation of *Klebsiella* can be suppressed by lactoferrin peptides; accordingly, its enrichment in the non-milk group may result from the absence of this lactoprotein-mediated inhibitory effect [48]. The *[Eubacterium] oxidoreducens* group has been reported to exert anti-inflammatory effects, modulate cholesterol homeostasis, and preserve gut barrier integrity via the biosynthesis of SCFAs [23].

Several limitations of this study should be acknowledged. First, this was a cross-sectional, single-center study involving community-dwelling adults recruited from one geographic location. Although this design reduced heterogeneity related to regional differences in diet, lifestyle, environmental exposure, and sample handling, it limited the generalizability of our findings and precluded causal or temporal inference regarding the associations between age, lifestyle factors, and gut microbiota composition. In addition, despite age stratification in the analyses, the broad age range of the study population may have introduced residual physiological and lifestyle-related confounding. Future multicenter prospective cohort studies or interventional trials involving geographically diverse populations are warranted to validate these associations. Furthermore, all correlation analyses were conducted using the combined study population rather than within individual age groups. Therefore, the observed associations may have been partially confounded by age-related differences.

Second, lifestyle information was obtained through self-reported questionnaires, which may have introduced recall bias. Although the questionnaire assessed the frequency of tea consumption, milk intake, and physical exercise, it did not collect detailed information regarding tea type, preparation method, milk type, fat content, consumption amount, exercise type, exercise intensity, or overall dietary patterns. Consequently, the potentially different associations of specific lifestyle behaviors with gut microbiota composition could not be evaluated.

Third, although participants who had used antibiotics, probiotics, prebiotics, or other microbiota-modulating medicines and health supplements within the previous four weeks were excluded, this washout period may not have been sufficient to eliminate their longer-term effects on gut microbiota composition and diversity. In addition, disease history and medication use were determined based on self-reported questionnaires and were not independently verified using medical or prescription records. Therefore, the possibility of unrecognized chronic diseases, unreported medication use, or residual effects of previous microbiota-modulating interventions, particularly among older participants, cannot be completely excluded.

## 5. Conclusions

In summary, age and lifestyle were associated with variations in gut microbiota composition. Older participants exhibited lower relative abundances of bacterial taxa previously reported to produce butyrate and higher abundances of several potentially pro-inflammatory genera. Tea consumption and regular exercise were associated with the enrichment of specific bacterial taxa previously reported to confer beneficial effects, whereas the associations between milk intake and gut microbiota were regulated by dosage and overall dietary context. Nevertheless, our findings should be interpreted in the context of the cross-sectional, single-center design and the limited taxonomic resolution of 16S rRNA gene sequencing. Collectively, our observations provide a basis for future mechanistic studies integrating metagenomic and metabolomic approaches to clarify the functional implications of these microbial alterations and validate their potential relevance to healthy aging. These results may further inform the development of microbiota-targeted strategies conducive to maintaining gut microbial homeostasis and promoting healthy aging.

## Figures and Tables

**Table 1:** Demographic and clinical characteristics of participants.

Category	YG (*n* = 44)	MG (*n* = 71)	EG (*n* = 44)	*p* Value
Age	29.5 (23–37.5)	52 (46–55)	69 (63.25–76.5)	-
Male ^#^	12 (27.3)	30 (42.3)	18 (40.9)	0.24 a
BMI ^#^				0.352 a
<18.5	0 (0)	0 (0)	0 (0)	
18.5–23.9	16 (29.6)	16 (16.3)	15 (26.8)	
24.0–27.9	14 (25.9)	15 (15.3)	12 (21.4)	
≥28.0	4 (7.4)	13 (13.3)	5 (8.9)	
NA	10 (22.7)	27 (38.0)	12 (27.3)	
Frailty phenotype score ^$^	1 (0–2)	1 (0.75–2)	1 (0–2)	0.631 b
GSRS *	23.32 ± 8.16	23.28 ± 6.93	23.74 ± 8.30	0.901 b
Tea drinkers ^#^	13 (24.1)	23(23.5)	8 (14.3)	0.055 a
Exercise group ^#^	17 (31.5)	22 (22.4)	27 (48.2)	0.004 a
Milk consumers ^#^	22 (40.7)	28 (28.6)	15 (26.8)	0.247 a

Note: ^#^ *n* (), ^$^ median (interquartile range), * mean ± standard deviation. a Statistics were based on Pearson’s chi-square test. b Statistics were based on the Kruskal–Wallis test. NA, missing BMI data (body mass index unavailable).

## Data Availability

The original contributions presented in this study are included in the article/Supplementary Material. Further inquiries can be directed to the corresponding author.

## Supplementary Materials

The following supporting information can be downloaded at https://www.mdpi.com/article/10.3390/microorganisms14081841/s1, Table S1: Characteristics and lifestyle information of participants assessed by questionnaire; Table S2: Differentially abundant gut microbial taxa associated with age groups; Table S3: Differentially abundant gut microbial taxa associated with tea consumption; Table S4: Differentially abundant gut microbial taxa associated with physical activity; Table S5: Differentially abundant gut microbial taxa associated with milk consumption.
