# Supplementary material for: Age-Related and Lifestyle-Related Differences in Gut Microbiota Composition Among Community-Dwelling Adults
Source: Microorganisms. 2026 Aug 19;14(8):1841. doi: 10.3390/microorganisms14081841 (PMC13515811; doi:10.3390/microorganisms14081841)
Supplement: Supplementary file 1 [file microorganisms-14-01841-s001.zip › Supplementary Table S1.pdf]

Supplementary Table S1. Characteristics and lifestyle information of participants assessed by que stionnaire.

Item	Response options
Age	______ years
Physical activity (exercise sessions >20 min)	□ No / very little (<1 time/week)<br>□ Yes (≥1 time/week)
Physical activity (exercise sessions >20 min)	□ No (<1 time/week)<br>□ Yes (≥1 time/week)
Habitual liquid milk consumption	□ No (<1 time/week)<br>□ Yes (≥1 time/week)

Participants were asked to report their habitual behaviors during the preceding 6 mont hs.
